# What Are We Protecting? On the Silent Arrival of *Anodonta cygnea* to Spain

**DOI:** 10.1002/ece3.71600

**Published:** 2025-10-09

**Authors:** Omar Sánchez, Sergio Quiñonero‐Salgado, Karl‐Otto Nagel, Joaquín López‐Soriano, Andrés Arias

**Affiliations:** ^1^ Department of Biology of Organisms and Systems (Zoology) University of Oviedo Oviedo Spain; ^2^ Associació Catalana de Malacologia (ACM), Museu Blau Barcelona Spain; ^3^ Malacological Section Senckenberg Research Institute and Natural History Museum Frankfurt/M Frankfurt am Main Germany; ^4^ Institute of Natural Resources and Territorial Planning (INDUROT) University of Oviedo Mieres Spain

**Keywords:** alien, conservation, freshwater mussels, native, reservoirs, Unionoida

## Abstract

Freshwater mussels play essential roles in ecosystem processes, such as water filtration and nutrient cycling, but, in the last decades, they have become increasingly threatened by habitat destruction, pollution, and the arrival of invasive species. In this study, a living population of the bivalve *Anodonta cygnea* is reported in Catalonia, Spain, based on molecular and morphological analyses. A comparison with *A. anatina* from a nearby population in southern France highlights the importance of using genetic tools to accurately distinguish between closely related freshwater mussel species. The introduction of *A. cygnea* likely reflects a recent range expansion, facilitated by human activities and environmental changes in the region. Its establishment in local ecosystems raises concerns about potential ecological impacts, including competition with native species and alteration of freshwater habitats. These findings underscore the critical need for effective monitoring and management strategies to mitigate the ecological risks associated with invasive species. This study emphasizes the importance of integrating molecular methods into conservation efforts to improve the understanding of species distributions and address the challenges posed by biodiversity loss in aquatic ecosystems.

## Graphical abstract

A living population of the bivalve *Anodonta cygnea* is reported in Catalonia, Spain, based on molecular and morphological analyses, that is, an integrative taxonomy approach. A comparison with a nearby population in southern France identified the latter as *A. anatina* , highlighting the importance of using genetic tools to accurately distinguish between closely related freshwater mussel species. The introduction of *A. cygnea* likely reflects a recent range expansion, facilitated by human activities and environmental changes in the region. Its establishment in local ecosystems raises concerns about potential ecological impacts, including competition with native species and alteration of freshwater habitats.

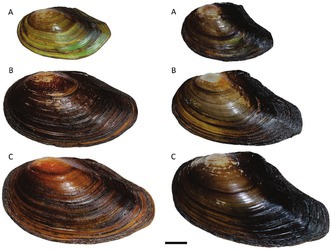

## 1 Introduction

Unionoidea Rafinesque, 1820 are a group of freshwater mussels whose populations are rapidly declining worldwide, bringing some of the species to the edge of extinction (Lopes‐Lima et al. 2017; Sousa et al. 2021). In the Iberian Peninsula, the dramatic situation of *Pseudunio auricularius* (Spengler, 1793) has led to specific programs oriented to its protection and the rescue of the remaining populations. However, the decline is also evident for some other species, such as *Potomida littoralis* (Cuvier, 1798) and *Anodonta anatina* (Linnaeus, 1758) (Rubio Millán et al. 2016; Nakamura 2022).

*Anodonta anatina* has a wide range extension, comprising most of Europe (Araujo et al. 2009; Welter‐Schultes 2012). In the NE Iberian Peninsula, particularly in Catalonia, a dramatic decline of its populations has been observed, with some restoration programs focused on the rescue of the remaining populations (Pou‐Rovira et al. 2017). Loss of suitable habitats, local extinction of fish hosts the mussel requires for their parasitic stage of their mussel larvae (glochidia), and the arrival of alien species are the main reasons for this decline (Nakamura 2022).

Two other Unionoidea with similar shell morphology have been recorded in northern Catalonia. *Sinanodonta woodiana* (I. Lea, 1834) is an invasive species that is rapidly expanding its range and displacing other native mussel species (Mehler et al. 2024). *Anodonta cygnea* (Linnaeus, 1758) is a species whose native status in the Iberian Peninsula has been debated by experts for decades (Nagel et al. 2022), being very difficult to identify due to its similar shell morphology to *A. anatina* . Therefore, all management programs of native Unionoidea should consider this difficulty in the identification of target species.

**FIGURE 1:**
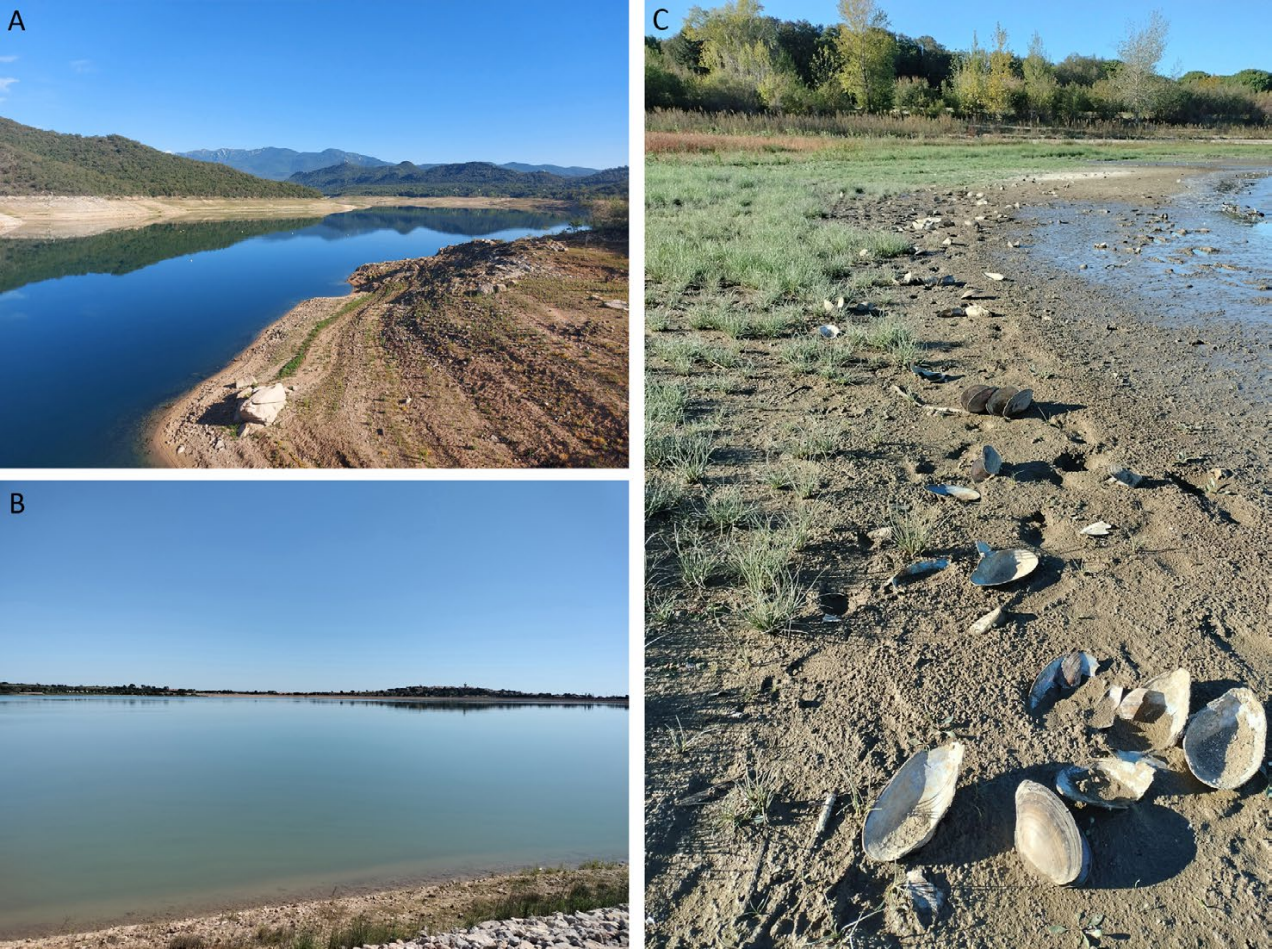
Habitats sampled in this study. (A) Overall view of the Darnius/Boadella reservoir (Girona province, Catalonia, Spain). The severe reduction in water levels is visible at the margins of the waterbody. (B) Lac de Villeneuve‐de‐la‐Raho (Pyrénées‐Orientales, France). (C) Dead shells of *Anodonta* in the shore of Lac de Villeneuve‐de‐la‐Raho.

The low water levels in some reservoirs in the northeast of the Iberian Peninsula during the very severe drought in 2022 and 2023 (down to 10%–15% of their maximum capacity) (www.embalses.net, see Figure 1) have facilitated the finding of freshwater bivalves at the shores. This included, for example, the first finding of the invasive *Corbicula fluminea* (O.F. Müller, 1774) in the upper part of the Muga river basin (Quiñonero Salgado and López Soriano 2023), which confirms this reservoir as a hotspot of aquatic alien species. During some sampling campaigns at different reservoirs in 2023, both dead and alive *Anodonta* specimens were also found in high abundances along the shore. Adults and juveniles were regularly found, suggesting a relatively healthy and reproductive populations, despite the drastic decrease in water levels in the reservoirs. This was very surprising, since *A. anatina* (presumably the only *Anodonta* species present) is now a rather rare species, with many of its populations rapidly vanishing in this whole area, so the possibility that they actually corresponded to a non‐native species should be considered.

With these antecedents, the objectives of this study are to present a morphological and genetic characterization of two Unionoidea mussel populations from two different reservoirs (one in North‐East Spain and one in the south of France); to determine the management consequences derived from the presence of these species; and to discuss the introduced and/or autochthonous status of these species in the Iberian Peninsula, as well as the potential pathways of introduction and their impacts.

## 2 Materials and Methods

### 2.1 Study Area, Specimen Collection, and Morphological Analysis

Dead and live specimens of *Anodonta* mussels were spotted on the shores of two different reservoirs, Darnius/Boadella reservoir (Alt Empordà, Girona province, Catalonia; 31 T 485600 4688800) and Lac de Villeneuve‐de‐la‐Raho reservoir (Pyrénées‐Orientales, south of France; 31 T 491200 4719800) in February 2024. The two reservoirs were chosen because of the presence of very high mussel densities. Dying specimens were collected for DNA analyses. Rivers and canals around the reservoirs were sampled, when possible, with little success, and no material was obtained for DNA analysis in these localities. Shells were tentatively identified following Araujo et al. (2009) and Reis et al. (2013).

### 2.2 DNA Extraction, PCR Amplification and Sequencing

For each population, two or three specimens were processed for DNA amplification and sequencing. The DNA was extracted from a small piece of foot tissue using the E.Z.N.A. Mollusk DNA Kit (Omega Bio‐Tek, Norcross, GA, USA). The mitochondrial cytochrome c oxidase subunit I (COI) gene fragment was amplified by polymerase chain reaction (PCR) in a total volume of 30 μL, using BR2/BF3 as primers (Elbrecht and Leese 2017). PCR conditions and mixture composition followed Martín‐Álvarez et al. (2024). The PCR product was checked via horizontal electrophoresis (2% agarose gel). Finally, the samples were sent for forward and reverse sequencing at MACROGEN (Madrid, Spain), using the standard Sanger sequencing method (Sanger and Coulson 1975).

### 2.3 Genetic Analysis

The forward and reverse sequences obtained by Sanger sequencing were edited for quality trimming, primer removal, and manual correction using Geneious Prime 2022.2.2 (https://www.geneious.com) and then aligned using ClustalW under default parameters. A consensus sequence was generated, and a preliminary genetic species identification was attempted using nBlast implemented in Geneious Prime. The phylogenetic analysis was conducted using five new sequences of *Anodonta* spp. and other 45 sequences from GenBank of the European *Anodonta* ( *A. anatina* , *A. cygnea* and *A. exulcerata* Porro, 1838) plus one sequence of *Margaritifera margaritifera* (Linnaeus, 1758) used as an outgroup. Sequences were deposited in GenBank with the accession numbers PQ351251–PQ351253 (Lac de Villeneuve‐de‐la‐Raho reservoir) and PQ351242–PQ351243 (Darnius/Boadella reservoir).

The test software IQ‐TREE v2.3.1 (Minh et al. 2020) was used to predict the nucleotide substitution model showing the best BIC scores using the ModelFinder option implemented in IQ‐TREE (Kalyaanamoorthy et al. 2017). A Maximum Likelihood tree was performed in IQ‐TREE v2.0 using Ultrafast Bootstrap options (10,000 bootstrap replicates) (Minh et al. 2013) and a search was conducted for the best scoring tree using the Hasegawa–Kishino–Yano model (HKY + F + I).

The PopART 1.7 program was used to obtain a haplotype network following the median‐joining model (Bandelt et al. 1999) from all the available sequences of the European populations of *A. cygnea* (*n* = 43).

## 3 Results

### 3.1 Shell Morphology

**FIGURE 2:**
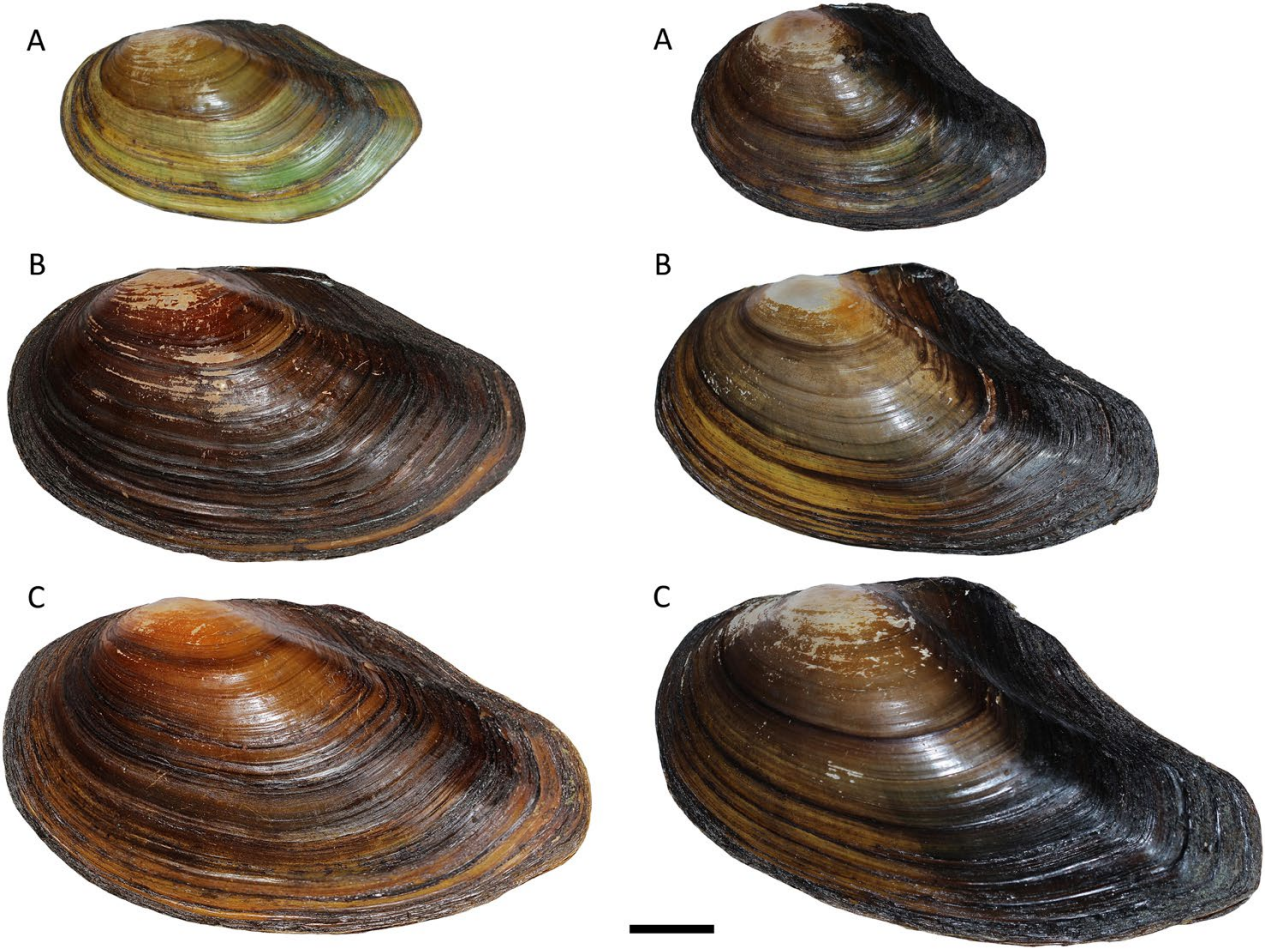
Specimens of *Anodonta cygnea* from Darnius/Boadella reservoir (Girona province, Catalonia, Spain) (left) in comparison with specimens of *Anodonta anatina* from Lac de Villeneuve‐de‐la‐Raho (Pyrénées‐Orientales, France) (right). Juvenile (A), subadult (B) and adult (C). Scale bar: 2 cm.

Overall shell shape of both populations was similar, although Villeneuve‐de‐la‐Raho specimens seem to have a concave upper posterior margin with a certain degree of variability (already commented by Araujo et al. 2009; Reis et al. 2013). They have a ridge on the posterior part of the shell, more evident in the juveniles (Figure 2A, right), and they present traces of an umbonal sculpture. In contrast, shells of the Boadella population (Figure 2, left) had a well‐marked sculpture on the umbo, characterized by concentric and discontinuous striae.

### 3.2 Molecular Assignment

The Blast identification engine identified the two sequences from Darnius‐Boadella reservoir (PQ351242–PQ351243) as *A. cygnea* with 99.19% to 100% of pairwise identity with 43 GenBank sequences. The remaining hits were with the species *A. exulcerata* and *Pseudanodonta complanata* (Rossmässler, 1835) with a similarity of only 91.80% or less. Additionally, the three sequences from the French population of Lac de Villeneuve‐de‐la‐Raho (PQ351251–PQ351253) were identified via Blast as *A. anatina* with 96.89% to 100% of pairwise identity with 348 sequences. The remaining hits were with the species *Anodonta* sp., *A. nuttalliana* (I. Lea, 1838), and *A. californiensis* I. Lea, 1852, with a similarity of only 90.72% or less.

**FIGURE 3:**
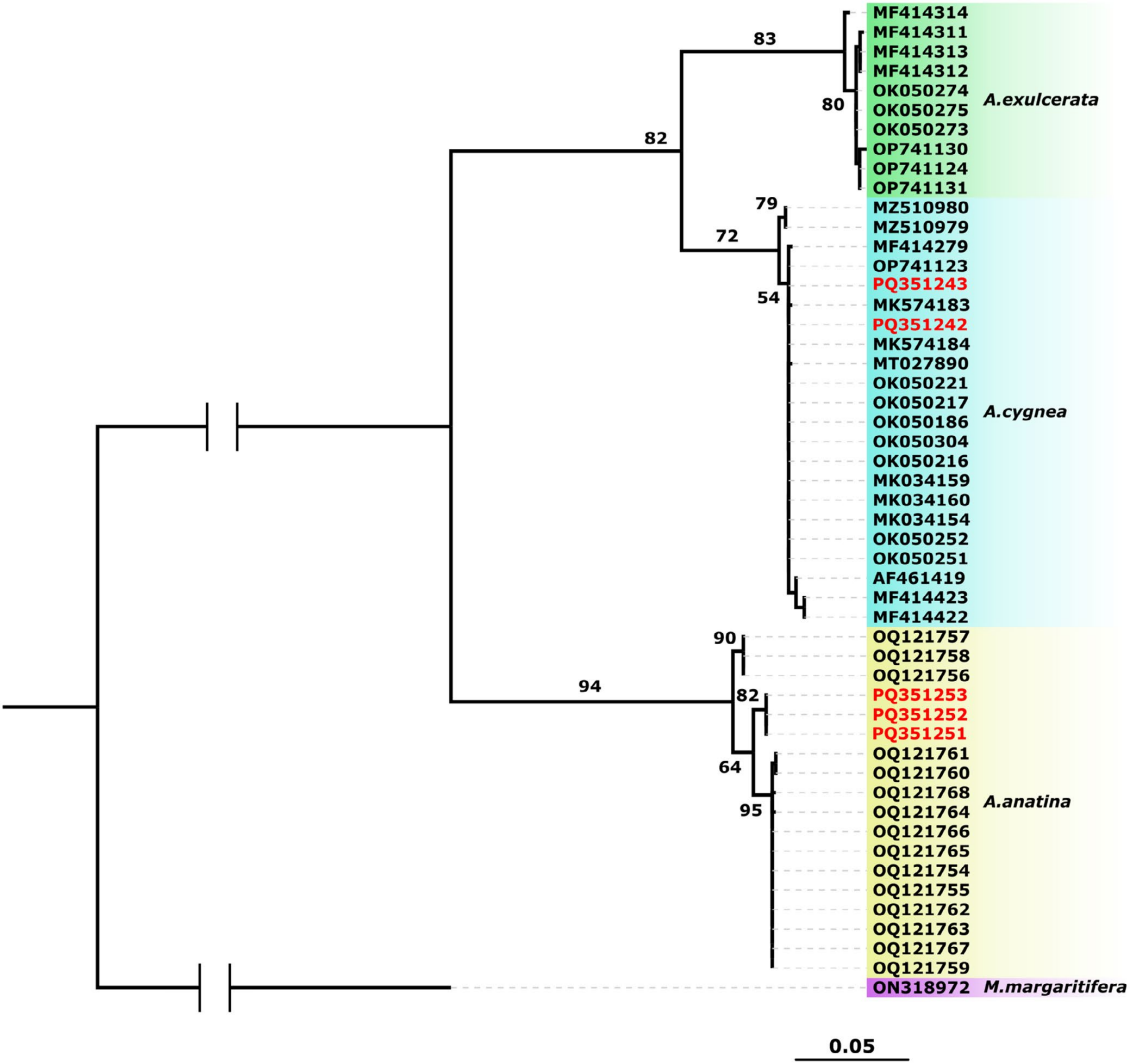
Maximum likelihood phylogeny inferred using the COI dataset in IQ‐TREE. Ultrafast bootstrap values are provided at relevant nodes. GenBank accessions numbers are listed adjacent to each scientific name. The new sequences are highlighted in red.

The phylogenetic analysis revealed that samples assigned to *A. cygnea* , as well as the sequences of *A. cygnea* from GenBank formed a well‐supported cluster with a high bootstrap support (i.e., 72%) (Figure 3). Likewise, the sequences assigned to *A. anatina* and those *A. anatina* sequences obtained from GenBank also formed a well‐supported cluster with a high bootstrap support (i.e., 84%) (Figure 3).

**FIGURE 4:**
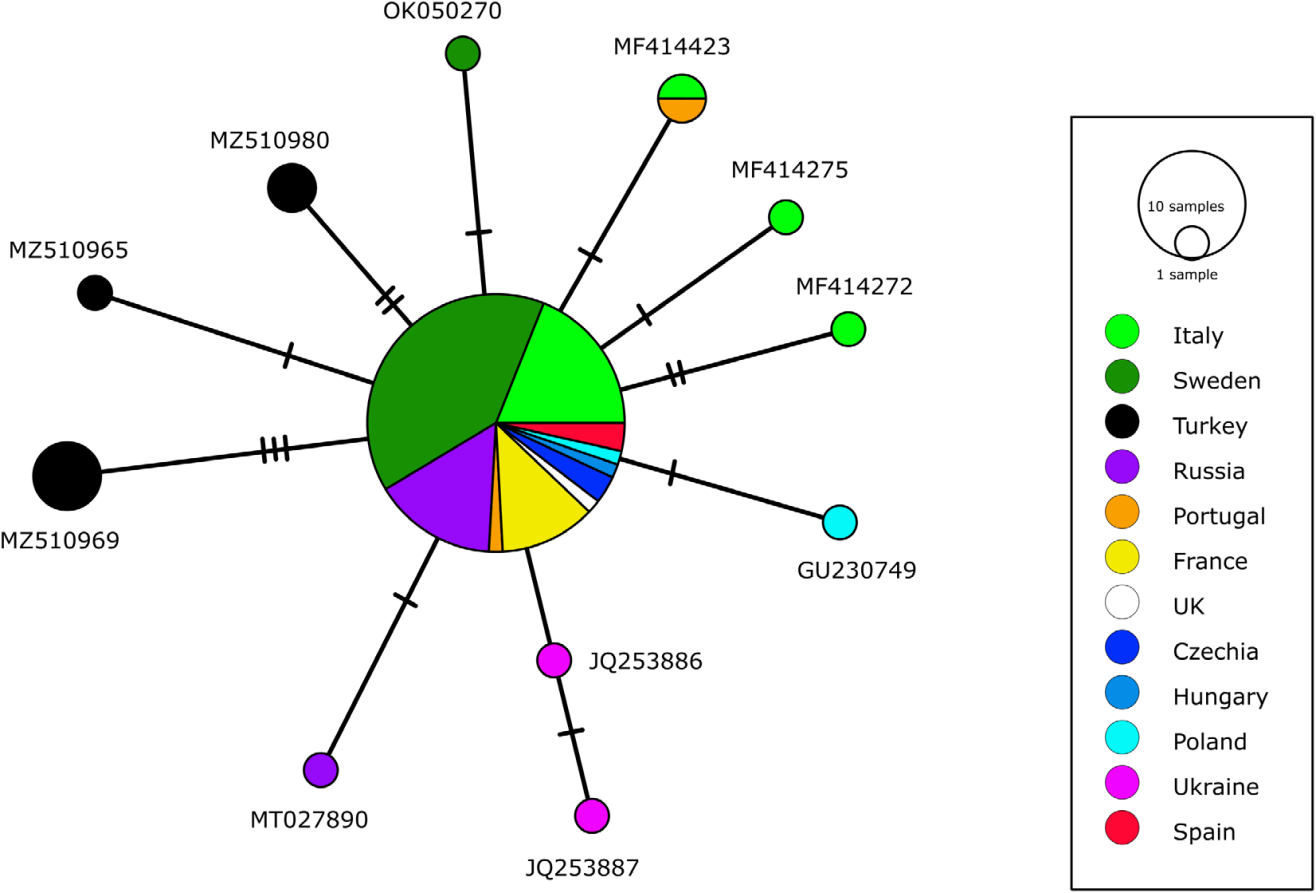
The mitochondrial haplotypes COI network of available sequences from European populations of *A. cygnea* . Node sizes are proportional to the number of sequences in which the haplotype was observed. Bars indicate the number of mutations needed to get from one haplotype to another, and the color of the circles indicate the country of origin of the *A. cygnea* individuals.

Haplotype analyses revealed the presence of a single haplotype for the two *A. cygnea* individuals studied (Figure 4). Both sequences have the same haplotype as most of the sequences available in GenBank from the different European countries. The haplotype network reveals little known European haplotypic diversity in *A. cygnea* , as the country with the most known haplotypes seems to be Italy with only four haplotypes followed by Turkey population with three haplotypes (Figure 4).

## 4 Discussion

The Darnius‐Boadella reservoir is located in an area rich in biodiversity, but also prone to invasions. A number of fishes have been cited for the first time in the Iberian Peninsula precisely in this reservoir, such as the common bream, *Abramis brama* (Linnaeus, 1758); the asp, *Leuciscus aspius* (Linnaeus, 1758); the perch, *Perca fluviatilis* (Linnaeus, 1758); the roach, *Rutilus rutilus* Linnaeus, 1758; the pikeperch, *Sander lucioperca* (Linnaeus, 1758); or the rudd, *Scardinius erythrophthalmus* (Linnaeus, 1758); as well as the spiny‐cheek crayfish, *Orconectes limosus* (Rafinesque, 1817) (Benejam et al. 2005, 2011; Clavero and García‐Berthou 2006; Merciai et al. 2018). Indeed, most of these introductions seem to come from France, given its close proximity to the border, to the point that its fish assemblage is even more similar to those of France than to those of the rest of the Iberian Peninsula (Clavero and García‐Berthou 2006).

*Anodonta anatina* has been previously recorded in this river basin (López Soriano and Quiñonero Salgado 2017; Pou‐Rovira et al. 2017), but it has been considered in severe regression, to the point that reintroduction efforts have been engaged in the context of a LIFE European project (Pou‐Rovira et al. 2017). By contrast, we provide the first confirmed occurence of *A. cygnea* for this river basin, and the second in Catalonia, after that of the Ebro River (Quiñonero Salgado et al. 2019; Nagel et al. 2022).

The French Lake was chosen as the second place for genetic study for three main reasons. First, a very dense population of *Anodonta* was observed, with a morphology of the shells suggestive of the presence of *A. cygnea* rather than *A. anatina* . Second, the small lake was full of the alien *Corbicula fluminea* (Müller, 1774), thus representing a rather ecologically deteriorated environment for a native bivalve to thrive in it. Finally, since *A. cygnea* has never been recorded from southern France, and given its geographical proximity, it could be a reasonable place of origin for a likely new population of this species in northern Catalonia.

Our genetic results confirm the presence of *A. cygnea* in the Muga River basin, an area where only *A. anatina* was previously reported. Although a few specimens were sequenced (because only recent dead individuals were collected due to the drought), not a single specimen of *A. anatina* was observed, and the shell morphology of the whole population suggests there is only one single taxon in this reservoir. In contrast, only *A. anatina* was found in the population studied in France (Lac de Villeneuve‐de‐la‐Raho, near Perpignan), although morphologically they were very similar to those from the Darnius/Boadella reservoir. More samples should be sequenced to confirm our findings. This observation confirms the need for DNA analyses to ensure a proper discrimination of these two species during routine examinations, even in different localities very close to each other, as morphological similarities may be misleading.

Few records of *A. cygnea* have indeed been reported in the whole Iberian Peninsula, and most of them could likely be misinterpretations of *A. anatina* specimens (Nagel et al. 2022). The present study represents the second population confirmed by DNA analysis, after the Portuguese population by Reis et al. (2013). It cannot totally be ruled out that the species could be native in the Peninsula, as there are some reports of conchologically confirmed *A. cygnea* specimens from different areas coming from historical collections (Portugal, Valencia, and Andalusia), which may indicate the presence of relict populations (Araujo et al. 2009; Nagel et al. 2022), but they could be the result of unnoticed recent introductions as well. Reis et al. (2013) confirmed the presence of a Portuguese population, and they suggested it could rather correspond to recent introductions (maybe from a century ago), as their haplotypes matched with other Central European populations. Consequently, our haplotypes seem to confirm this hypothesis.

It is possible that *A. cygnea* has started a recent colonization (or re‐colonization, if it was actually autochthonous) in the recent decades in NE Iberian Peninsula, likely from populations from Central Europe, but not necessarily from the south of France. To investigate a likely French origin, we obtained specimens from the huge population of *Anodonta* located some 30 km away from Darnius/Boadella, in the Lac de Villeneuve‐de‐la‐Raho (Pyrénées‐Orientales). Here, individuals of very big size and at very high densities were observed, despite the small surface of the lake, its eutrophication, and the abundance of the alien *C. fluminea* (see Figure 1C). Despite their similar morphology to the Darnius/Boadella population, their sequences unequivocally show them to be *A. anatina* .

The recent records of *A. cygnea* in Ebro River (Quiñonero Salgado et al. 2019), although still not confirmed by DNA analyses, and now this new population very close to the Spanish/French border, suggest the species may have been introduced in the last decades in NE Iberia. Further genetic and detailed analyses are needed in other locations to confirm this spread, and an eventual replacement of the native *A. anatina* in different hydrological basins, including the Ebro River, or even the presence of mixed populations of both species.

Sport fishing may be the reason for these new introductions, as both rivers (Ebro and Muga) are intensively used for this activity, with particular presence of many users coming from different European countries, which may have favored this likely recent importation, as stowaways in fishing materials. A correlation between the highly infested areas for exotic fish and the presence on *A. cygnea* is quite evident. The scarcity of the native Unionoidea, some of them critically endangered in Iberia (Lopes‐Lima et al. 2017; García‐Álvarez 2019; Nakamura 2022) may have also contributed to the fast adaptation and expansion of *A. cygnea* in empty niches which are still optimal for this species. Particularly, the species seems to thrive in rather lentic ecosystems, particularly reservoirs, as is the new case reported in this paper, or in highly modified rivers, such as the Ebro River where it was already reported.

It is likely that in the coming years more populations of *A. cygnea* will be found in the Iberian Peninsula, which may confirm a fast expansion in this territory. An eventual displacement of *A. anatina* populations should be carefully monitored, while *A. cygnea* continues to expand its populations. Notably, all the management efforts regarding the protection of *A. anatina* should be carefully reviewed, since most of them could be unwittingly targeting a non‐native species. Ongoing work by the authors is indeed oriented to this purpose.

## Author Contributions

**Omar Sánchez:** conceptualization (equal), data curation (equal), formal analysis (equal), methodology (equal), software (equal), writing – original draft (lead), writing – review and editing (equal). **Sergio Quiñonero‐Salgado:** conceptualization (equal), formal analysis (equal), methodology (equal), resources (equal), writing – original draft (equal), writing – review and editing (lead). **Karl‐Otto Nagel:** conceptualization (equal), formal analysis (equal), methodology (equal), resources (equal), writing – original draft (equal), writing – review and editing (lead). **Joaquín López‐Soriano:** conceptualization (equal), formal analysis (equal), methodology (equal), resources (equal), writing – original draft (equal), writing – review and editing (lead). **Andrés Arias:** conceptualization (equal), funding acquisition (lead), investigation (equal), supervision (lead), writing – original draft (equal), writing – review and editing (lead).

## Conflicts of Interest

The authors declare no conflicts of interest.

## Data Availability

All the genetic data is available at GenBank using the accession numbers provided in Figures 3 and 4.
